# Anticonvulsant Effect of Carbenoxolone on Chronic Epileptic Rats and Its Mechanism Related to Connexin and High-Frequency Oscillations

**DOI:** 10.3389/fnmol.2022.870947

**Published:** 2022-05-09

**Authors:** Benke Liu, Xiao Ran, Yanjun Yi, Xinyu Zhang, Hengsheng Chen, Yue Hu

**Affiliations:** ^1^Department of Neurology, Children’s Hospital of Chongqing Medical University, Chongqing, China; ^2^Ministry of Education Key Laboratory of Child Development and Disorders, Chongqing Key Laboratory of Pediatrics, National Clinical Research Center for Child Health and Disorders, China International Science and Technology Cooperation Base of Child Development and Critical Disorders, Chongqing, China; ^3^Shenzhen Baoan Women’s and Children’s Hospital, Jinan University, Shenzhen, China

**Keywords:** carbenoxolone, chronic epilepsy, connexin, ripples, fast ripples

## Abstract

**Objective:**

This study was designed to investigate the influence and mechanism of gap junction carbenoxolone (CBX) on dynamic changes in the spectral power of ripples and fast ripples (FRs) in the hippocampus of chronic epileptic rats.

**Methods:**

The lithium-pilocarpine (PILO) status epilepticus (SE) model (PILO group) and the CBX pretreatment model (CBX + PILO group) were established to analyze dynamic changes in the spectral power of ripples and FRs, and the dynamic expression of connexin (CX)26, CX32, CX36, and CX43 in the hippocampus of chronic epileptic rats.

**Results:**

Within 28 days after SE, the number of spontaneous recurrent seizures (SRSs) in the PILO group was significantly higher than that in the CBX + PILO group. The average spectral power of FRs in the PILO group was significantly higher than the baseline level at 1 and 7 days after SE. The average spectral power of FRs in the PILO group was significantly higher than that in the CBX + PILO group at 1, 7, and 14 days after SE. Seizures induced an increase in CX43 expression at 1 and 7 days after SE, but had no significant effect on CX26, CX36, or CX32. CBX pretreatment did not affect the expression of CXs in the hippocampus of normal rats, but it inhibited the expression of CX43 in epileptic rats. The number of SRSs at 2 and 4 weeks after SE had the highest correlation with the average spectral power of FRs; the average spectral power of FRs was moderately correlated with the expression of CX43.

**Conclusion:**

The results of this study indicate that the energy of FRs may be regulated by its interference with the expression of CX43, and thus, affect seizures. Blocking the expression of CX43 thereby reduces the formation of pathological high-frequency oscillations (HFOs), making it a promising strategy for the treatment of chronic epilepsy.

## Introduction

There are extensive high-frequency oscillations (HFOs) in neural networks. The term HFOs refers to electroencephalogram (EEG) activity with a frequency of 40–500 Hz, including γ oscillation (40–80 Hz), ripple oscillation (80–200 Hz), and fast ripple oscillation (250–500 Hz) (Höller et al., 2019). HFOs can be divided into physiological and pathological, which differ in their frequency, location and mechanisms (Le Van Quyen et al., 2006). Physiological HFOs gradually mature with brain development. Ripples (>140 Hz) are observed in the hippocampus of rats during their second week after birth. A vitro hippocampal model has confirmed that physiological HFOs can be controlled by a feedback circuit between GABAergic interneurons and pyramidal cells (Hájos and Paulsen, 2009). In physiological states, HFOs are associated with sensory information processing and hippocampal memory function (Höller et al., 2019); whereas, in pathological states, HFOs are closely related to epilepsy (Frauscher et al., 2017; Velmurugan et al., 2019; Tingley and Buzsáki, 2020).

Our previous study showed that the energy of HFOs in hippocampal regions CA1 and CA3 of the lithium-pilocarpine (PILO) status epilepticus (SE) model was significantly higher than that of physiological HFOs (Song et al., 2016). The spectral power of fast ripples (FRs) during seizures can be used as a quantitative indicator providing an early warning of seizures (Song et al., 2016).

The generation mechanism of HFOs is not completely clear; HFOs may be caused by a variety of mechanisms rather than a single mechanism of neural cells and networks. At present, it is believed that pathological HFOs, whether ripples or FRs, mainly reflect the action potential of principal cells. It may be related to the gap junction (GJ) network (Jiruska et al., 2017). Gap junctions are intercellular channels composed of special transmembrane proteins. These transmembrane protein families are called connexins (CXs) (Beyer and Berthoud, 2018). At present, more than 20 kinds of CXs have been found, and CX26 is expressed in a variety of nerve cells, especially neurons (Su et al., 2017); CX32 is widespread on oligodendrocytes (Men et al., 2019). CX36 is a connexin preferentially expressed by neurons, and it plays an important role in the transmission of electrical signals (Li et al., 2019). CX43 has the highest expression in astrocytes and it may be involved in the regulation of nerve injury, as well as epileptogenesis (Deshpande et al., 2017). The increase in electrical coupling between GJs transforms physiological HFOs into pathological HFOs (Stacey et al., 2011), which induce synaptic plasticity (Jefferys et al., 2012). Our previous study confirmed that in an acute epilepsy rat model, the spectral power of FRs and the degree of seizures can be downregulated by inhibiting the expression of different CXs (Ran et al., 2018).

It is not known whether connexins participate in the occurrence of pathological HFOs, thereby establishing abnormal electrosynaptic transmission and increasing susceptibility to seizure in the chronic phase of epilepsy. Therefore, we established an chronic epilepsy rat model to observe dynamic changes in the energy of HFOs and the expression of CXs in the hippocampus, to explore the role of GJ receptor blockers in the chronic phase of epilepsy, and to clarify whether GJ plays a key role in the occurrence and development of epilepsy, in order to provide a theoretical basis for the selection of new targets for antiepileptic therapy.

## Materials and Methods

**FIGURE 1:**
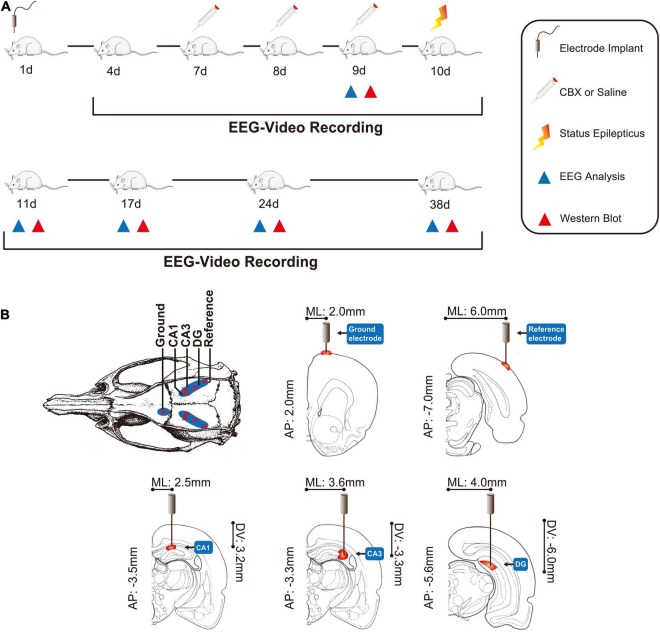
Comprehensive modeling of chronic epilepsy. **(A)** Schematic representation of the overall protocol and various analyses performed in chronic epileptic rats. **(B)** The positions of the EEG recording electrodes. CBX, carbenoxolone; AP, anteroposterior; ML, mediolateral; DV, dorsoventral.

The overall protocol and time-course of the evaluation of various outcomes are shown in Figure 1A.

### Establishment of the Epilepsy Model and Electroencephalogram Recordings

This study was approved by the Ethics Committee of Chongqing Medical University. The ethical approval number is 2020135. Adult male Sprague–Dawley rats weighing 180–220 g were obtained from the Animal Research Institute of Chongqing Medical University. Before surgery, all the rats were intra-peritoneally (IP) injected with penicillin (1 ml/kg, 160,000 U/ml) to prevent intracranial infection. The rats were then anesthetized with 10% chloral hydrate (2 ml/kg, IP) and immobilized in a stereotaxic frame (Shenzhen Reward Life Science Company, Shenzhen, China) to surgically implant the microelectrode (nichrome wires, 0.1 mm in diameter). The recording electrode was implanted as follows: CA1-AP: 3.3–3.7 mm from bregma; ML: 2.0–3.0 mm and DV: 3.0–3.5 mm from the surface of the neocortex; and CA3–AP: 3.3 mm, ML: 3.5–3.7 mm, DV: 3.0–3.5 mm; DG–AP: 5.6 mm, ML: 4.0 mm and DV: 6.0 mm. The reference electrode was implanted on the surface of the neocortex of the bilateral parietal lobe (AP: 7.0 mm; ML: 6.0 mm), and the left forehead was used as the ground (AP: 2.0 mm; ML: 2.0 mm) (Song et al., 2016; Ran et al., 2018). The positions of these electrodes are shown in Figure 1B (Bao and Shu, 1991; Zhuge, 2005). Dental cement (Shanghai Medical Equipment Limited by Share Ltd., Shanghai, China) was used to fasten the microelectrode to the skull. After surgery, the rats were housed individually in cages under standard conditions, in a controlled environment (23 ± 2°C, 50–55%) under a 12 h:12 h light/dark cycle (lights on at 08:00 h), and *ad libitum* access to food and water.

Normal intracranial EEG signals (EEG 1200 systems, 32 channels, Nihon Kohden Corporation, Tokyo, Japan) were recorded on the 3rd day after surgery for 5 days (5–8 h/day), and the lithium-pilocarpine SE model was established on the 9th post-operative day (Ran et al., 2018). The rats were injected with pilocarpine (50 mg/kg IP, Sigma, Canada) 18–20 h after an injection of lithium chloride (127 mg/kg IP, Sigma, Canada). If generalized seizures (stage 4 of Racine’s criteria) were not elicited within 30 min, a second injection of pilocarpine (10 mg/kg, IP) was administered. SE was defined as the presence of continuous generalized seizures for at least 60 min without returning to normal behavior between seizures. Atropine sulfate (1 mg/kg IP, Shanghai and Feng pharmaceutical companies, Shanghai, China) was injected to limit peripheral cholinergic effects 10 min after the injection of pilocarpine. SE was arrested using diazepam (10 mg/kg IP, Shanghai Asahi Dongpu Pharmaceutical Co. Ltd., Shanghai, China) (Song et al., 2016; Ran et al., 2018). After successful modeling, the rats were injected intraperitoneally with glucose saline (2 ml/day for 3 days: 1 ml 10% glucose + 1 ml 0.9% sterile saline) to reduce mortality after SE. At 24 h after SE, EEG signals were recorded for 28 consecutive days. The sampling frequency was 1 kHz, high pass 0.16 Hz, and low pass 500 Hz. Rat seizures were monitored and the number of spontaneous recurrent seizures (SRSs) was recorded.

Forty rats were randomly divided into the lithium-pilocarpine status epileptic model (PILO group) and the carbenoxolone (CBX) pretreatment model (CBX + PILO group), with 20 rats in each group. The PILO group was pretreated with saline [8 ml/kg dose for 3 consecutive days (8:00 am and 8:00 pm) by intraperitoneal injection] before the PILO injection, the CBX + PILO group was injected with CBX [50 mg/kg dose for 3 consecutive days (8:00 am and 8:00 pm) by intraperitoneal injection] before the PILO injection. Of these, 14 rats in the PILO group and 13 rats in the CBX + PILO group were successfully modeled. Due to factors, such as shedding of implanted electrodes and the death of rats, the EEG signals with SRSs of 11 rats in the PILO group and 9 rats in the CBX + PILO group were recorded over 28 consecutive days. One experimenter randomly coded the two groups of rats, and the other experimenter selected 8 rats in each group as the research subjects, using a random coding lottery.

### Electroencephalogram Analysis

We selected 10-min samples of the electrical activity of EEG signals for quantitative analysis at 5 time points (1 day before SE, and 1, 7, 14, and 28 days after SE). Quantitative analysis was conducted on the HFOs for the 10 min EEG signals collected at each of the above time points. In this study, the Morlet wavelet algorithm was used to extract ripples and FRs signals, including average and peak spectral power analysis (Song et al., 2016; Ran et al., 2018). The average spectral power of ripples and FRs refers to the average of all spectral power in the entire observation time window; the peak spectral power of ripples and FRs refers to the spectral power at a certain time point in the entire observation time window, which is the highest spectral power of the observation time window (Song et al., 2016; Ran et al., 2018). The leads where changes in the spectral power of ripples and the FRs were the most significant were defined as responsibility leads (RLs) (Song et al., 2016; Ran et al., 2018).

### Western Blot

The rats were sacrificed at 5 time points (1 day before SE, and 1, 7, 14, and 28 days after SE). Western blot was used for the semi-quantitative analysis of CX26 (Sigma-Aldrich, United States; diluted 1:400 dilution), CX32 (Sigma-Aldrich, United States; 1:400), CX36 (Sigma-Aldrich, United States; 1:600), and CX43 (Sigma-Aldrich, United States; 1:8000) expression in the hippocampus. Immunoreactive bands were visualized by the ECL Advance Western blot reagent (Bio-Rad, United States). The optical densities of the immunoreactive bands were quantified by densitometry using Labworks 4.6 software (EC3 Imaging System, UVP Inc., United States). The relative levels of the CX26, CX32, CX36, and CX43 were expressed as ratios (CX26/β-actin, CX32/β-actin, CX36/β-actin, and CX43/β-actin).

### Statistical Analysis

SPSS version 23.0 was used for the statistical analysis. Quantitative data are expressed as mean ± SD. A two-way repeated-measures ANOVA was used for the statistical analysis of HFOs’ spectral power and connexin expression between the groups, and *t*-tests were used for pairwise comparisons. Pearson’s correlations were used to analyze other quantitative data. A bad value refers to a maximum or minimum value that does not conform to the normal distribution. A *p* < 0.05 was considered to be a statistically significant difference.

## Results

### Behavioral Study

We did not observe any abnormal behaviors during the 18–24 h after lithium chloride injection. All the rats exhibited peripheral cholinergic effects after the pilocarpine injection, including pupil narrowing, piloerection, hemolacria, diarrhea, and wet-dog shakes. Seizures in all the rats were classified as stages IV and V, and all the rats exhibited SE; the peripheral cholinergic effects gradually disappeared after atropine injection. After diazepam injection, the seizures stopped.

**FIGURE 2:**
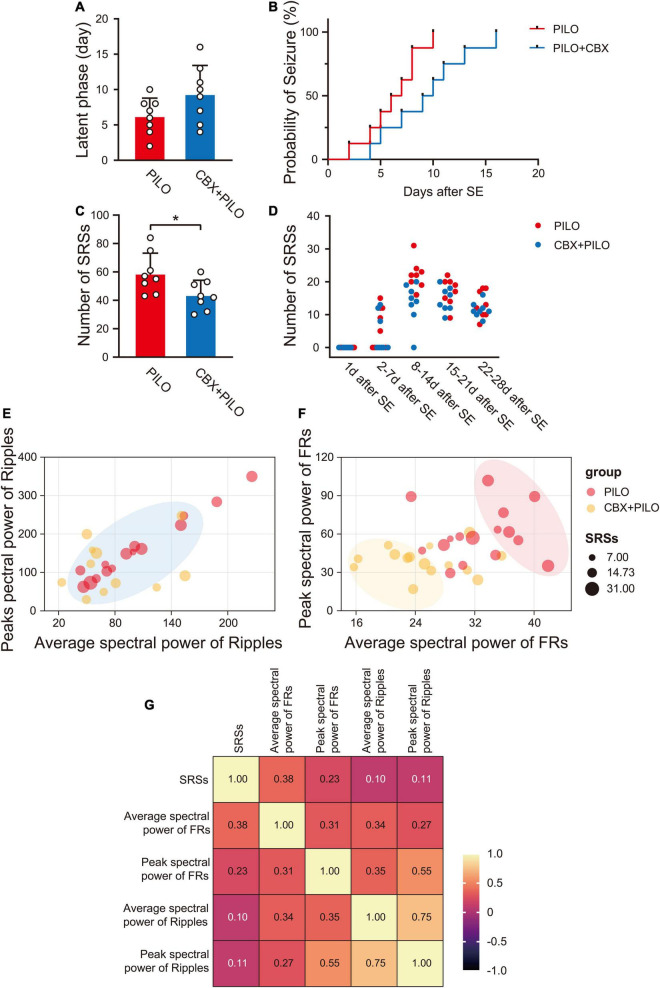
Lithium-pilocarpine produced chronic SRSs in rats. **(A)** Average latent phase in PILO and CBX + PILO groups. **(B)** Incidence curve of epileptic rats over time, showing latent phase to first seizure after SE. **(C)** Average number of SRSs in PILO and CBX + PILO groups during the 4 weeks after SE. **(D)** The number of SRSs per week after SE in PILO and CBX + PILO groups. **(E,F)** Bubble diagram of the relationship between the number of SRSs and the spectral power of ripples/FRs at 2 and 4 weeks after SE in PILO and CBX + PILO groups, the gathered bubbles of the two groups means weak correlation, and the separated bubbles of the two groups means strong correlation. **(G)** The correlation coefficient matrix of the number of SRSs and the spectral power of HFOs at 2 and 4 weeks after SE (average spectral power of FRs, *r* = 0.38; peak spectral power of FRs, *r* = 0.23; peak spectral power of ripples, *r* = 0.11; average spectral power of ripples, *r* = 0.10). *P < 0.05; SRSs, spontaneous recurrent seizures; PILO, pilocarpine; SE, status epilepticus; FRs, fast ripples; HFOs, high-frequency oscillations.

After a latent phase lasting approximately 2–10 days (6.25 ± 2.55 days), during which no organized activity was recorded, spontaneous grade I-V SRSs reappeared in the rats in the PILO group, showing no statistical difference from rats in the CBX + PILO group [4–16 days (9.38 ± 4.03 days), *t*(14) = 1.852, *p* = 0.085] (Figure 2A). However, the probability of a seizure in the CBX + PILO group was lower than that in the PILO group (Figure 2B). Within 4 weeks, the average number of SRSs in the PILO group was significantly higher than that in the CBX + PILO group [58.88 ± 14.32 vs. 43.75 ± 10.39, *t*(14) = 2.418, *p* = 0.03] (Figure 2C). The number of SRSs per week after SE in the two groups are shown in Figure 2D.

The relationship between the number of SRSs at 2 and 4 weeks after SE and the spectral power of ripples/FRs in the PILO and CBX + PILO groups are shown in Figures 2E,F; the bubbles of the two groups that are gathered in Figure 2E are separated in Figure 2F. The number of SRSs at 2 and 4 weeks after SE had the highest correlation with the average spectral power of FRs (*r* = 0.38, *p* = 0.035). Then, the correlations decreased with the peak spectral power of FRs (*r* = 0.23, *p* = 0.213), the peak spectral power of ripples (*r* = 0.11, *p* = 0.593), and the average spectral power of ripples (*r* = 0.10, *p* = 0.621) (Figure 2G).

### Quantitative Analysis of High-Frequency Oscillations

**FIGURE 3:**
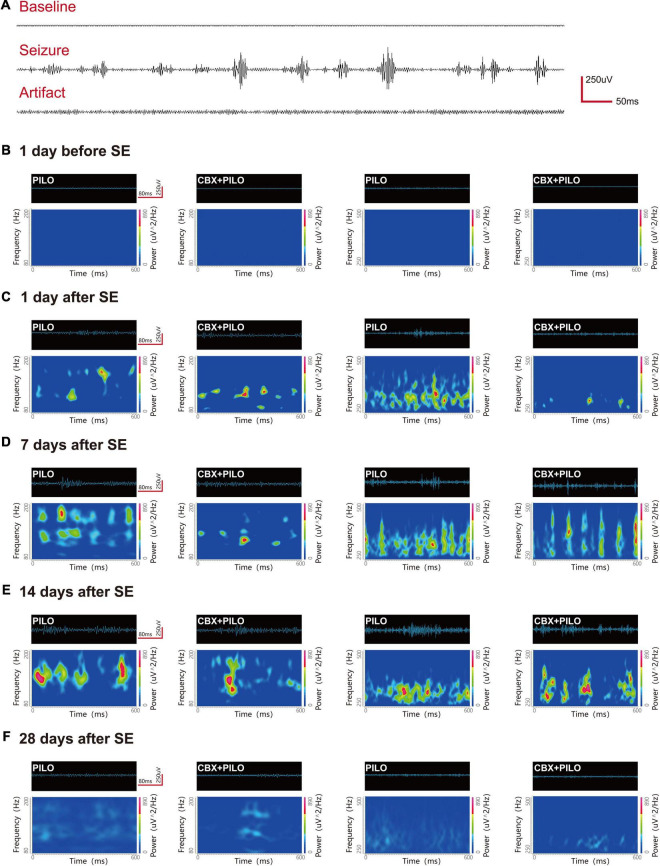
Waveforms and spectrograms features of lithium-pilocarpine-induced electrographic seizures. **(A)** Representative EEG traces of the baseline, epileptic seizure, and an artifact. **(B–F)** Waveforms and spectrograms showing the spectral and temporal characteristics of ripples (80–200 Hz) and FRs (250–500 Hz) in the PILO and CBX + PILO groups at 5 time points [1 day before SE, and 1, 7, 14, and 28 days after SE]. The waveforms were filtered with a band pass filter of 80–200 Hz and 250–500 Hz. The spectrograms reflect the accumulated time-frequency representations of the corresponding waveforms. The data demonstrate that the initiation of seizures was associated with an increase of HFOs energy, and that pretreatment with CBX could reduce HFOs energy and the degree of seizures. The HFOs are characterized by rhythmic bursts, which are significantly different from systematic artifacts, such as power line noise and its harmonics.

Before modeling, ripples and FRs were observed in hippocampal regions CA1, CA3, and DG in normal rats. The most significant dynamic changes in the energy of HFOs were noted in the CA1 and CA3 regions (*n* = 6) and the DG region (*n* = 2) in the PILO group, and the CA1 and CA3 regions (*n* = 8) in the CBX + PILO group. Waveforms and spectrograms showing the spectral and temporal characteristics of lithium-pilocarpine-induced electrographic seizures are shown in Figure 3. Further statistical analyses of the average and peak spectral power of ripples and FRs are shown in Tables 1, 2.

**TABLE 1:** Dynamic changes in the average and peak spectral power of ripples recorded in RLs in the PILO and CBX + PILO groups at different time points after SE (*n* = 8).

Time points	Average spectral power of ripples		Peak spectral power of ripples	
	PILO	CBX + PILO	PILO	CBX + PILO
1 day before SE	97.75 ± 39.83*	86.99 ± 27.98^#^	139.73 ± 62.89^#^	121.37 ± 37.28*
1 day after SE	108.06 ± 41.1	79.14 ± 54.16*	195.2 ± 69.96*	107.62 ± 55.37^a,#^
7 days after SE	112.23 ± 66.33	71.16 ± 32.95	206.14 ± 129.92	98.51 ± 58.74
14 days after SE	102.53 ± 61.59	75.66 ± 49.9^#^	161.56 ± 92.73	116.69 ± 77.09
28 days after SE	94.85 ± 50.5	76.03 ± 40.89^#^	157.44 ± 77.47*	105.61 ± 41.4^#^
*F*	0.141	0.128	0.636	0.189
*P*	0.965	0.971	0.641	0.942

*^a^p < 0.05 compared to the PILO group at the same time.*

*Eliminated a bad value at this time point.

*^#^Eliminated two bad values at this time point.*

*RLs, responsibility leads; PILO, pilocarpine; CBX, carbenoxolone; SE, status epilepticus.*

**TABLE 2:** Dynamic changes in the average and peak spectral power of FRs recorded in RLs in the PILO and CBX + PILO groups at different time points after SE (*n* = 8).

Time points	Average spectral power of FRs		Peak spectral power of FRs	
	PILO	CBX + PILO	PILO	CBX + PILO
1 day before SE	29.64 ± 6.75	32.19 ± 9.14	55.81 ± 16.67	58.08 ± 26.48
1 day after SE	52.19 ± 9.19^b^	24.74 ± 4.36^a^	93.15 ± 44.93^b^	45.43 ± 20.85^a,^*
7 days after SE	51.93 ± 5.99^b^	25.27 ± 6.05^a,^*	90.44 ± 20.28*	40.1 ± 24.12^a^
14 days after SE	34.15 ± 6.27	25.85 ± 4.02^a^	67.6 ± 23.1	38.4 ± 10.59^a^
28 days after SE	31.05 ± 3.85	25.4 ± 7.31	51.26 ± 15.43	37.24 ± 13.25
*F*	23.27	1.309	4.127	1.461
*P*	<0.001	0.286	<0.05	0.236

*^a^p < 0.05 compared to PILO group at the same time.*

*^b^p < 0.05 compared to 1 day before SE in the same group.*

*Eliminated a bad value at this time point.

*^#^Eliminated two bad values at this time point.*

*FRs, fast ripples; RLs, responsibility leads; PILO, pilocarpine; CBX, carbenoxolone; SE, status epilepticus.*

There was no significant difference in the average spectral power of ripples in the PILO and CBX + PILO groups before and after SE, nor was there a significant difference in the average spectral power of ripples between the two groups at any time point (*p* > 0.05) (Table 1).

The average spectral power of FRs in the PILO group at 1 and 7 days after SE was significantly higher than that at baseline [*F*(4,35) = 23.27, *p* < 0.05], peaking 1 day after SE, then gradually decreasing to baseline until 28 days after SE. There was no significant difference in the average spectral power of FRS in the CBX + PILO group before and after SE (*p* > 0.05) (Table 2). The average spectral power of FRs in the PILO group was significantly higher than that in the CBX + PILO group at 1, 7, and 14 days after SE [*F*(4,69) = 16.31, *p* < 0.05] (Table 2).

The dynamic changes in the average and peak spectral power of ripples and FRs were similar (Tables 1, 2).

### Expression of Connexins

**FIGURE 4:**
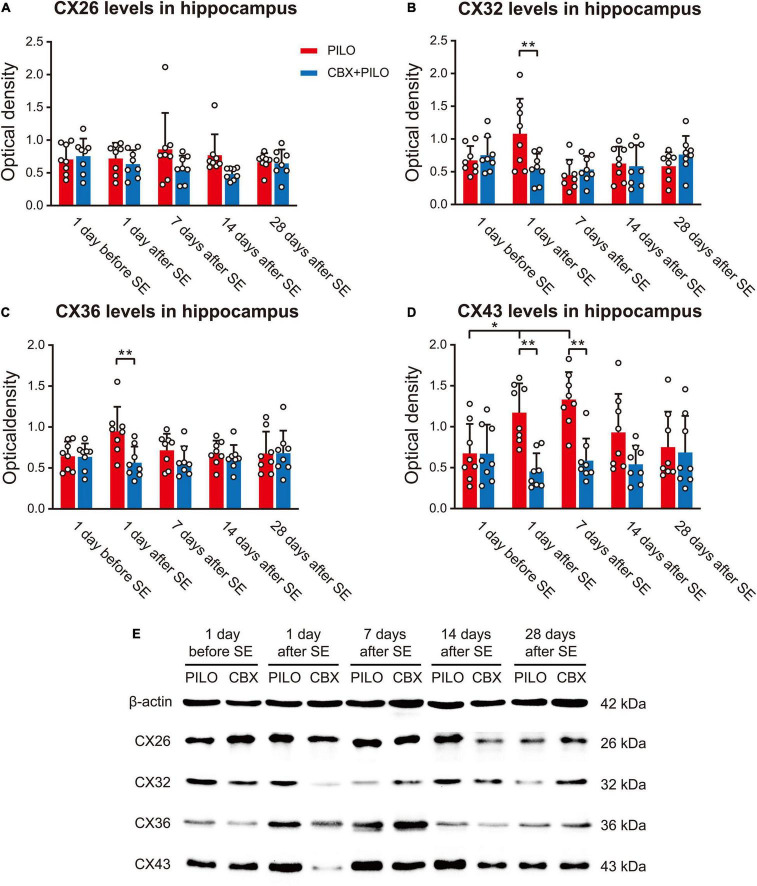
Expression of four kinds of CXs. **(A–D)** Western blot showing the expression of CX26, CX32, CX36, and CX43 in the hippocampus before and after SE in the PILO and CBX + PILO groups. **(E)** β-actin is used as a loading control. Each bar represents the mean ± SD of eight separate assays. *P < 0.05, **P < 0.01; CX, connexin.

There was no significant difference in the expression of CX26 in the PILO and CBX + PILO groups before and after SE, nor was there a significant difference in the expression of CX26 between the two groups at any time point (Figure 4A).

There was no significant difference in the expression of CX32 and CX36 before and after SE in the PILO and CBX + PILO groups, but the expression of CX32 [*t*(14) = 2.614, *p* = 0.02] and CX36 [*t*(14) = 3.075, *p* < 0.05] in the PILO group was significantly higher than that in the CBX + PILO group 1 day after SE (Figures 4B,C).

Compared to the expression of CX43 before SE in the PILO group, CX43 expression increased significantly at 1 and 7 days after SE [*F*(4,35) = 3.63, *p* < 0.05], and then returned to baseline. There was no significant difference in the expression of CX43 in the CBX + PILO group before and after SE. The expression of CX43 in the PILO group at 1 and 7 days after SE was significantly higher than that in the CBX + PILO group [*F*(4,70) = 5.83, *p* < 0.05] (Figure 4D). The dynamic expression of four kinds of CXs in the hippocampus of chronic epileptic rats are shown in Figure 4E.

### Correlation Analysis of High-Frequency Oscillations and Connexins

**FIGURE 5:**
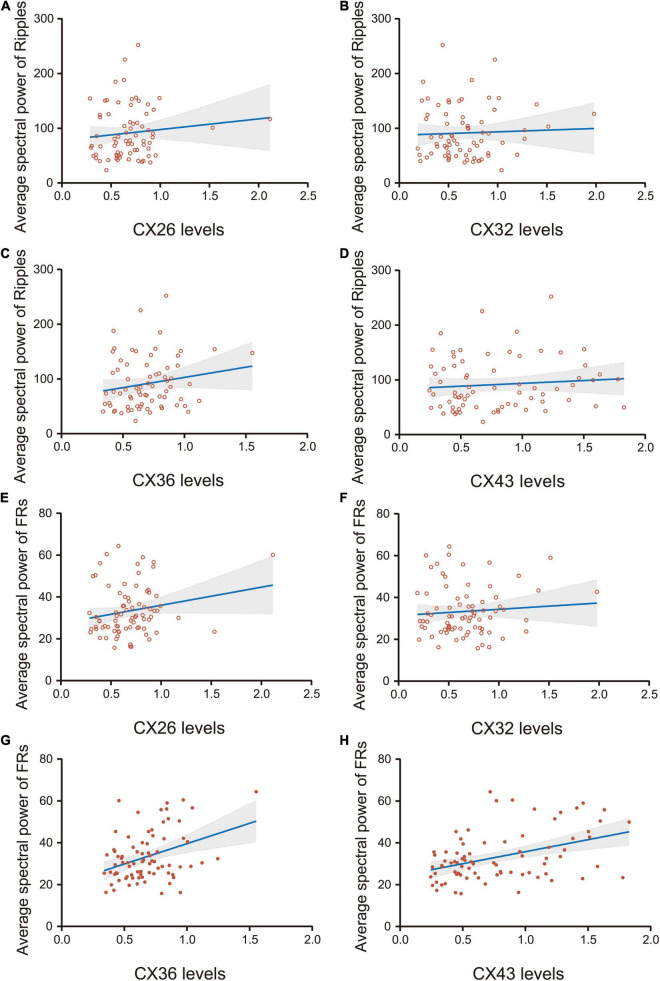
Correlation analysis of the average spectral power of HFOs and the expression of CX26, CX32, CX36, and CX43. **(A–D)** There was no significant correlation between the average spectral power of ripples and the expression of CX26, CX32, CX36, or CX43 (*P* > 0.05). **(E,F)** There was no significant correlation between the average spectral power of FRs and the expression of CX26 or CX32 (*p* > 0.05). **(G)** The average spectral power of FRs was weakly correlated with CX36 expression (*r* = 0.37, *p* < 0.05) and **(H)** moderately correlated with CX43 expression (*r* = 0.42, *p* < 0.05).

There was no significant correlation between the average spectral power of ripples and the expression of CX26, CX32, CX36, or CX43 (*p* > 0.05) (Figures 5A–D). There was no significant correlation between the average spectral power of FRs and the expression of CX26 or CX32 (*p* > 0.05) (Figures 5E,F). The average spectral power of FRs was weakly correlated with CX36 expression (*r* = 0.37, *p* < 0.05; Figure 5G), and moderately correlated with CX43 expression (*r* = 0.42, *p* < 0.05; Figure 5H).

## Discussion

Pilocarpine is a post-ganglionic cholinergic drug, which can directly stimulate M-cholinergic receptors to produce a cholinergic effect, thereby inducing seizures by activating central cholinergic receptors. The adult rat lithium-pilocarpine epilepsy model exhibits three periods: an acute phase–6–24 h after SE; a latent phase–no organized activity and EEG for at least 1 day; and a chronic phase–the appearance of SRSs. SRSs are important clinical manifestations of epilepsy. In the rat model of epilepsy, which is created using lithium-pilocarpine, SRSs appear after SE. The damage and manifestations of this model are similar to human temporal lobe epilepsy, and this model is one of the most commonly used experimental epilepsy models (Song et al., 2016). In this study, SRSs occurred within 16 days after SE, indicating entry into the chronic phase of epilepsy in the experimental rats.

Enhanced intercellular GJ communication may be involved in epileptic production (Mylvaganam et al., 2014). CBX is a glycyrrhizonic acid derivative that reduces plasma membrane mobility and inhibits gap junction conductance through multiple pathways, including protein kinase, G protein, transport ATP enzyme, and CX phosphorylation (Walrave et al., 2020). It is a broad-spectrum GJ blocker that acts on a variety of CXs. Whether in cell culture *in vitro*, acute brain slices, or *in vivo* animal experiments, CBX has been proven to block GJ quickly and reversibly, thus, further reducing the number of spontaneous/induced seizures, and reducing the duration, frequency, and amplitude of epileptic discharges (Vincze et al., 2019; Walrave et al., 2020). CBX can reduce the frequency of spontaneous HFOs activity (Naggar et al., 2020). This study confirmed that CBX can reduce the number of SRSs and the average spectral power of FRs in rats, with an antiepileptic effect.

High-frequency oscillations in the human brain are affected by sleep. Studies show that the rate of HFOs is highest during non-rapid eye movement (NREM) sleep and lowest during rapid eye movement (REM) sleep and the waking stage; the area of HFOs during NREM sleep is larger (von Ellenrieder et al., 2017). Our previous study showed that physiological and pathological HFOs have similar sleep balance characteristics (Yan et al., 2021). At present, the correlation between HFOs and sleep in the animal brain is not clear. Therefore, in our study, we selected 10-min samples of EEG signals during the interictal period (waking stage) to explore the effect of CBX on the energy changes of ripples and FRs in rats with chronic epilepsy.

High-frequency oscillations are generated by multiple mechanisms such as synchronized inhibitory post-synaptic potentials with sparse pyramidal cell firing or principal cell action potentials (Ylinen et al., 1995; Bragin et al., 2011). Synchronization of fast firing within the population of interconnected neurons leads to the formation of an episode of high-frequency population spikes, which is extracellularly recorded as an HFO event. It requires synchronization on a millisecond time scale, which is achieved *via* fast synaptic transmission or non-synaptic mechanisms like gap-junction coupling or ephaptic interactions–a synchronizing mechanism that depends on specific geometric organization and tight cellular arrangement (Jiruska et al., 2017). Both pyramidal neurons and interneurons are involved in HFO generation, but pyramidal cells fire preferentially at the highest amplitude of the ripples, and interneurons begin to discharge earlier than the pyramidal cells do (Cepeda et al., 2020). The inhibitory effect of the interneurons is maintained in ripples (Jefferys et al., 2012), whereas FRs reflect hypersynchronous population spikes of excitatory pyramidal cells (Kanazawa et al., 2015). The formation mechanism of HFOs with different frequencies may be different. The ripples may be formed by the synchronization of the inhibitory post-synaptic potential generated by excitatory neurons mediated by GABAA receptors, while the FRs may be derived from the field potential formed by transient, highly synchronized and sudden discharges of excitatory neurons with pathological connections (Jacobs et al., 2014; Frauscher et al., 2017). Our previous studies confirmed that the frequency of HFOs is not necessary to distinguish physiological and pathological states, that ripples and FRs can be present in both the normal and the epileptic hippocampus, and that the main difference between the two states is the different energy of HFOs (Ran et al., 2018). This study found that during the chronic phase of epilepsy, the average and/or peak spectral power of ripples did not significantly change before and after SE in the PILO and CBX + PILO groups. However, the average and peak spectral power of FRs in the PILO group were significantly higher at 1 and 7 days after SE than those 1 day before SE, and that the average spectral power of FRs in the PILO group were significantly higher than those in CBX + PILO group at 1, 7, and 14 days after SE. This suggests that in the chronic phase of epilepsy, FRs respond more to the real situation of SRSs than ripples do. In a study of intracranial EEG in epileptic patients, González Otárula et al. (2019) found the distribution of ripples was widespread, while FRs were restricted to the epilepsy initiation region. The association of FRs with seizure onset zone may be stronger than that of ripples (Frauscher et al., 2018). Animal studies suggest that ripples are related to physiological functions, such as the formation of memory and cognition, while FRs are related to the seizure onset zone (Nevalainen et al., 2020). FRs are able to influence the specific synaptic driving function of CA1 pyramidal cells, make neuronal firing randomized, and ultimately lead to the loss of control of selective discharges in the hippocampus (Valero et al., 2017). It has been suggested that an energy analysis of FRs may be a more sensitive and specific biomarker of epilepsy, compared to ripples.

Gap junction is considered to be an important component of the neuronal network, with synchronized neuronal activity and field potential oscillations (Posluszny, 2014). The EEG signals during the seizure and interictal seizure phases are correlated with the degree of electrical coupling to the GJ (Roopun et al., 2010). The upregulation of CX43 by the transient receptor potential vanoxalate-4 (TRPV4) may be involved in the pathophysiological process of epilepsy (Men et al., 2019), and the specific CX43 mimic peptide and TAT-Gap19 can reduce spontaneous seizures by inhibiting the function of GJ channels/hemi-channels of CX43 between astrocytes (Delvaeye et al., 2018; Walrave et al., 2018). We investigated the effects of seizures and CBX intervention on the expression of different CXs in the hippocampus in chronic epilepsy and found that seizures induced an increase in CX43 expression at 1 and 7 days after SE, but had no significant effect on CX26, CX36, or CX32. CBX pretreatment did not affect the expression of CXs in the hippocampus of normal rats, but it inhibited CX43 expression in epileptic rats. Further analyses revealed that the expression of CX43 was more strongly correlated with the spectral power change of FRs than the other three CXs, suggesting that the energy of FRs may be regulated by interfering with the expression of CX43, so as to affect seizures. Therefore, in addition to traditional antiepileptic drugs, drugs should be developed that target blocking the electrical conduction of GJ, thereby reducing pathological HFOs formation, which can provide a new strategy for treating epilepsy. However, the causal relationship between the energy of FRs and the expression of CX43 is still unclear. What specific signaling pathways of connexin regulate HFOs, or how neuroelectrical activity affect connexin expression, remain to be explored in the future.

## Data Availability Statement

The raw data supporting the conclusions of this article will be made available by the authors, without undue reservation.

## Ethics Statement

The animal study was reviewed and approved by Ethics Committee of Chongqing Medical University.

## Author Contributions

YH conceived and designed the experiments and revised the manuscript critically for important intellectual content. BL made acquisition and interpretation of data and was involved in drafting the manuscript. BL, XR, and YY performed the experiments. XZ and HC performed data analysis. All authors wrote the manuscript and approved the submitted version.

## Conflict of Interest

The authors declare that the research was conducted in the absence of any commercial or financial relationships that could be construed as a potential conflict of interest.

## Publisher’s Note

All claims expressed in this article are solely those of the authors and do not necessarily represent those of their affiliated organizations, or those of the publisher, the editors and the reviewers. Any product that may be evaluated in this article, or claim that may be made by its manufacturer, is not guaranteed or endorsed by the publisher.
